# Diagnosis and treatment of transnasal endoscopic optic canal decompression for traumatic optic neuropathy

**DOI:** 10.3389/fnins.2023.1168962

**Published:** 2023-05-16

**Authors:** Xiang Tu, Cheng Xiong, Hui Qi, Yangming Ou, Jing Rao, Yueqi Sun, Yunping Fan, Guiqin Liu

**Affiliations:** ^1^Department of Otolaryngology, The Seventh Affiliated Hospital, Sun Yat-sen University, Shenzhen, China; ^2^The Department of Orbital Disease and Ophthalmic Oncology, Shenzhen Eye Hospital, Shenzhen Eye Institute, Jinan University, Shenzhen, China

**Keywords:** nasal endoscopy, optic nerve decompression, traumatic optic neuropathy, trauma, glucocorticoid

## Abstract

**Objective:**

To investigate the clinical efficacy and prognostic factors of transnasal endoscopic optic decompression in the treatment of traumatic optic neuropathy (TON).

**Methods:**

A retrospective analysis was performed on 13 TON patients in The Seventh Affiliated Hospital of Sun Yat-sen University and Shenzhen Eye Hospital in Shenzhen City (China) from June 2020 to April 2022. These patients had received transnasal endoscopic optic decompression, and hormonal and neurotrophic drugs were given after surgery. Visual acuity (VA) improvement was used as the criterion to judge clinical efficacy.

**Results:**

In a total of 13 patients, 13 injured eyes (12 men and 1 woman; mean age, 28.0 ± 11.8 years) received transnasal endoscopic optic decompression. After surgery, nine patients had improved VA, whereas four patients failed to show any improvement, resulting in a total effective rate of 69.2%. Of the six patients with no light perception preoperatively, three had effective results after the operation, giving an effective rate of 50.0%. Of the seven patients with residual light sensation preoperatively, six had effective results after the operation, giving an effective rate of 85.7%. Of the 10 patients operated on within 7 days after injury, seven had effective results, giving an effective rate of 70%. Of the three patients injured and operated on after 7 days, two had effective results, giving an effective rate of 66.7%.

**Conclusion:**

Transnasal endoscopic optic nerve decompression is an effective treatment method for TON. The presence of residual light perception and the timing of surgery within 7 days are crucial to the prognosis.

## Introduction

Traumatic optic neuropathy (TON) is a type of injury to the optic nerve resulting from external force impacting the optic nerve canal in the skull or face. This condition is characterized by a sudden or gradual loss of visual acuity (VA) and/or visual field defects following trauma (Yu-Wai-Man, 2015). TON is caused mainly by an external force acting on the optic nerve that causes optic nerve damage. The basic injury mechanism of this disease can be divided into direct and indirect injury. Direct injury is caused mostly by open craniomaxillofacial trauma, which causes anatomical fracture and avulsion of the optic nerve directly (Yu-Wai-Man and Griffiths, 2013). Direct injury can lead to severe irreversible vision loss or even complete loss, and the treatment response and prognosis are poor. TON caused by indirect injury, which is transmitted to the optic nerve by the energy generated though open or closed craniomaxillofacial trauma, causes the fracture of the osseous optic canal or the compression of the surrounding structures, such as the reactive edema of the optic nerve sheath, indirectly. The vascular supply and neurotrophic supply of retinal ganglion cells (RGCs) might be compromised to trigger apoptosis. The primary and secondary injuries caused by RGC apoptosis also lead to axon transport disorders, inflammatory reactions, and electrochemical disorders, which eventually lead to optic nerve injury (Giacci et al., 2014). However, owing to the complex pathophysiological mechanism of TON, various related molecular pathogenesises still need to be further explored. Treatment options for TON are complex, with some studies suggesting that glucocorticoid pulse or transnasal endoscopic optic nerve decompression might be crucial in preserving surviving RGCs (Yu-Wai-Man and Griffiths, 2013; Sefi-Yurdakul and Koç, 2018; Yu et al., 2020), Despite this, some studies advocate for a conservative approach of observation, because spontaneous visual improvement can occur in some TON patients (Sosin et al., 2016). There is still much debate among medical professionals about the most effective treatment for TON owing to a lack of large-scale clinical evidence. In recent years, advancements in clinical research and technology have led to improved diagnosis and treatment strategies for TON, with transnasal endoscopic decompression surgery gaining widespread recognition (Abhinav et al., 2015). However, various factors can impact the outcome of TON treatment, such as the timing of surgery, preoperative vision, and optic nerve tube fractures (Ma et al., 2018). This study analyzed the clinical data of 13 TON patients retrospectively, and evaluated the factors influencing surgical outcomes to determine surgical efficacy on TON.

## Patients and methods

We collected clinical data from patients with TON who were admitted to the Department of Otorhinolaryngology of the Seventh Affiliated Hospital of Sun Yat-sen University and the Department of Orbitopathy and Ophthalmic Oncology of Shenzhen Eye Hospital (China) between June 2020 and April 2022. The Ethics Committees of the two hospitals did not require the application of ethical approval for this type of study, and all aspects of the study were conducted in accordance with the Declaration of Helsinki. In the 13 TON patients, four of whom had a surgical interval of >4 days and also accepted hormone treatment before the operation, whereas the other nine patients accepted no treatment before surgery. To assess the effect of optic nerve decompression, we first conducted ophthalmological examinations to assess the visual acuity of patients, which helps to rule out preexisting optic neuropathy and retinopathy. The best corrected VA (BCVA) was tested initially using a Snellen chart, and a VA of <0.01 was documented with counting fingers (*CF*), hand motion (HM), light perception (LP), and no light perception (NLP). Additionally, ophthalmic equipment inspection, such as optical coherence tomography (OCT) (Carl Zeiss Meditec, United States or Heidelberg Engineering, Germany), visual field (Carl Zeiss, Germany Humphrey HFA), visual evoked potential (ROLAND, Germany), anterior segment photography (CSO, Italy), and fundus photography, were used to appraise visual function. Second, image analysis, such as computed tomography (CT)/magnetic resonance imaging (MRI), was applied to observe the optic canal fracture and orbital fracture. A thin-slice CT scan of the orbit was performed, and CT angiography (CTA) was performed in the case of craniocerebral trauma. Third, complete routine preoperative examination for patients was carried out. Finally, all patients underwent ophthalmological examinations after transnasal endoscopic optic nerve decompression and had postoperative drugs, such as hormones, nutritional nerves, and vasodilators. Efficacy determination: this study divided visual acuity into five different grades: I, NLP; II, LP; III, HM; IV, *CF*; and V, logarithm of minimum angle of resolution (logMAR) with a VA of 0.02 and above. Postoperative VA that improved by one level or more than preoperative was defined as valid; no improvement in VA was defined as ineffective; a reduction of one level or more was defined as a failed operation.

## Results

### Demographic characteristics

There were 13 patients (12 males, 1 female, average age 28.0 ± 11.8 years) with 13 injured eyes (12 right eyes, 1 left eye). The causes of injury were six car accidents, four blunt object injuries, and three fall injuries, and none of them had simultaneous bilateral optic nerve injury. Of these, six patients had no light sensation (I) before surgery, three had LP (II), one had *CF* (IV), and three had preoperative residual VA (V) (Table 1).

**Table 1:** Clinical features for 13 patients.

Case	Age at onset(years)	Involved eye	Time to operation (days)	Cause of injury	Fracture of skull base	Preoperative vision	Postoperative vision for 6 months
1/M	12	OD	0.83	BFT	N	NLP	0.6
2/M	40	OS	1	TA	Y	NLP	NLP
3/M	47	OD	1	FI	Y	NLP	NLP
4/M	30	OD	0.71	TA	N	NLP	LP
5/M	18	OS	10	TA	N	LP	*CF*
6/M	23	OD	30	TA	N	0.16	0.25
7/M	26	OD	4	BFT	N	0.1	0.4
8/M	13	OD	14	BFT	N	0.05	0.05
9/M	41	OD	7	FI	N	LP	*CF*
10/M	24	OS	1	TA	N	LP	*CF*
11/F	29	OD	3	BFT	N	*CF*	0.3
12/M	42	OD	1	TA	N	NLP	NLP
13/M	19	OD	0.67	FI	Y	NLP	0.4

BFT, blunt force trauma; CF, counting fingers; D, days; F, female; FI, falling injury; HM, hand motion; LP, Light perception; M, male; N, no; NLP, no light perception; OD, oculus dexter; OS, oculus sinister; TA, traffic accident; Y, yes.

### Clinical characteristics

**Figure 1:**
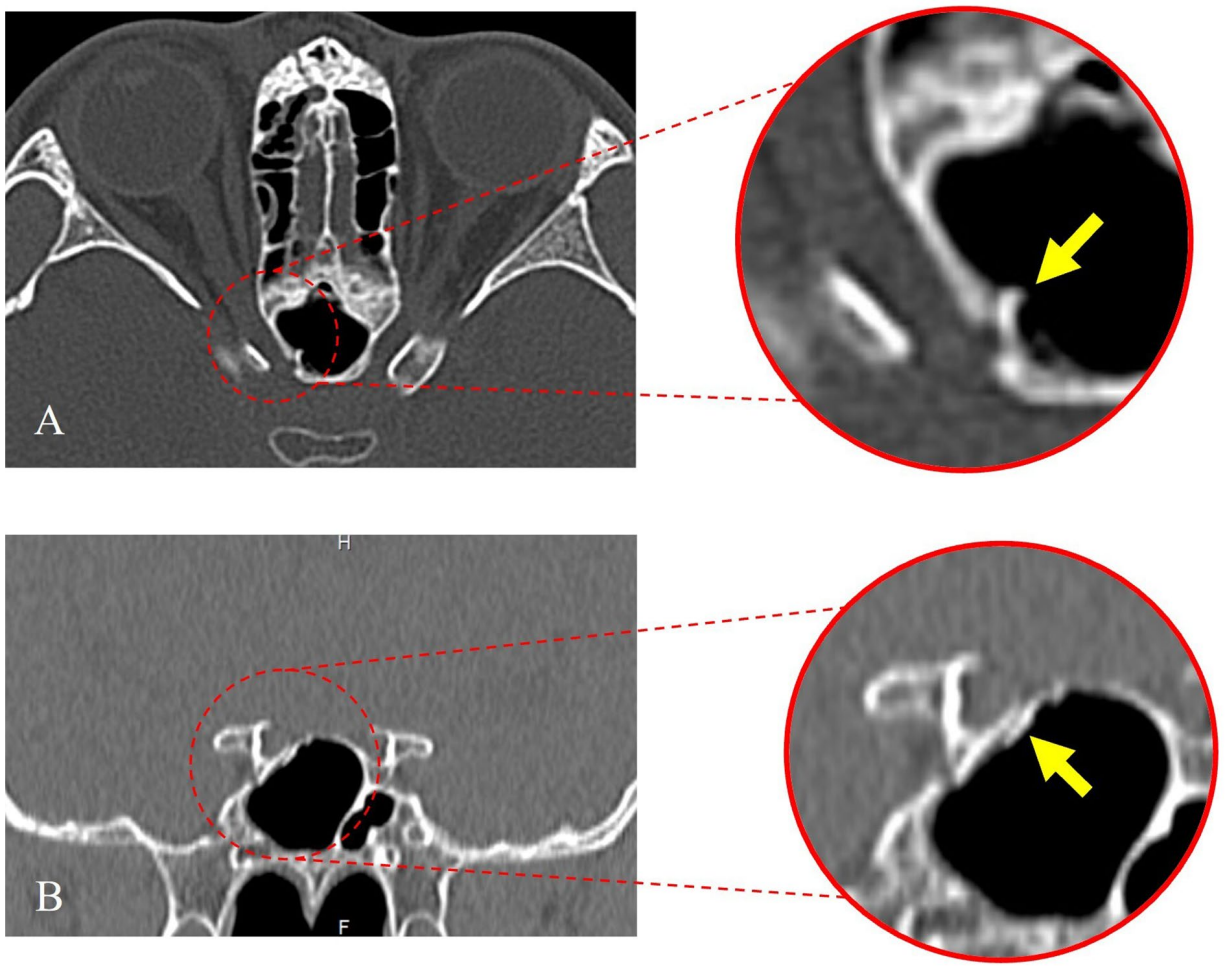
Preoperative orbital computed tomography (CT) thin-slice scan. **(A)** The axial position of the optic nerve tube shows the fracture line. **(B)** The coronal position of the optic nerve tube shows the fracture line.

**Figure 2:**
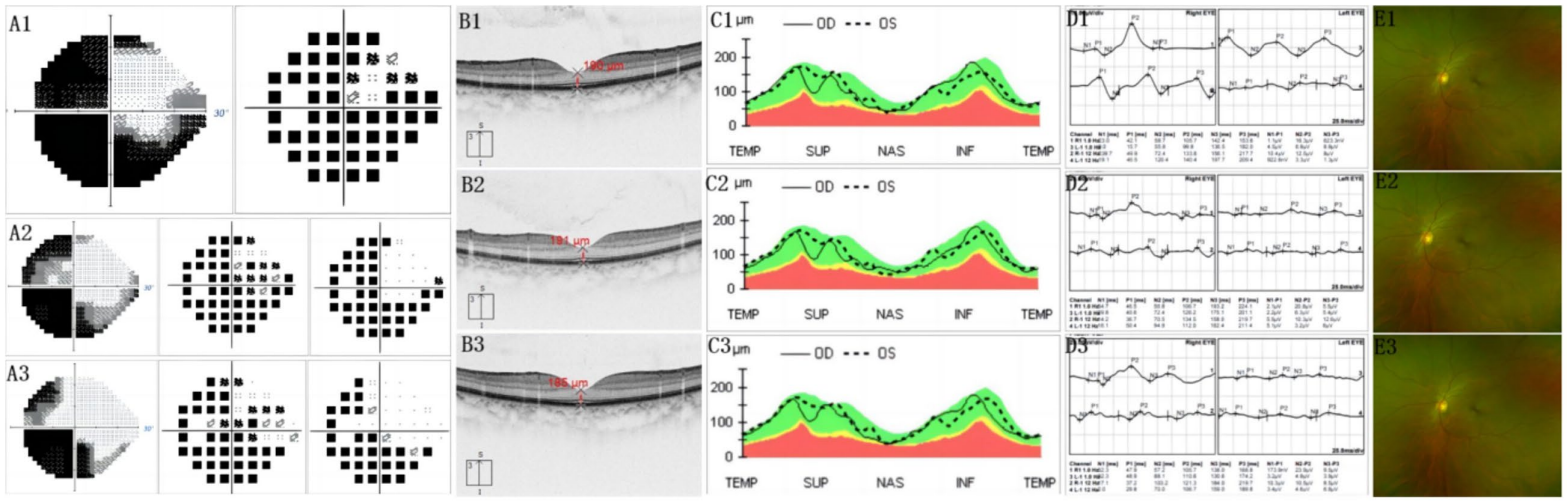
**(A1)** Before surgery, most of the visual field of the injured eye disappeared. Only visible is a small part of the temporal visual field. The pattern deviation chart shows that the threshold of average defect is exceeded. **(A2)** First day after operation, temporal visual field improved significantly. **(A3)** At 6 months after surgery, temporal visual field expanded. Also, a small amount of nasal visual field appeared. **(B1.2.3)** Indicates that the fovea of the macula and thickness are normal. **(C1.2.3)** Shows that the thickness of the retinal nerve fiber layer was normal. **(D1)** Before surgery, the amplitude of N2, P2, and N3 descended apparently in the left eye, compared with the right eye. **(D2)** On the first day after surgery, the amplitude of N2, P2, and N3 had not obviously improved, compared with before surgery. **(D3)** At 6 months after surgery, the amplitude of N2，P2, and N3 had no obviously improved, compared with the first day after surgery. **(E1.2.3)** Implies that fundus blood vessels were normal and the retina is flat, without bulge. **(B1,C1,E1)** Before surgery. **(B2,C2,E2)** First day after surgery. **(B3,C3,E3)** At 6 months after surgery.

Figure 1 demonstrates the fracture line of the optic nerve tube from the horizontal and coronal positions, respectively. The fracture was located in the right sphenoid sinus and was accompanied by a fracture of the nasal bone (Figure 1). The visual field of the patient was improved on the first day after the operation and improved significantly 6 months after the operation (Figure 2). The fovea of the macula and thickness were normal (Figures 2B1−B3), as was the thickness of the retinal nerve fiber layer (Figures 2C1−C3). Prior to surgery, the amplitude of N2, P2, and N3 had decreased apparently in the left eye compared with the right eye (Figure 2D1), whereas, on the first day post surgery, the amplitude of N2, P2, and N3 had not improved obviously compared with the preoperative surgery (Figure 2D2). By 6 months after the surgery, the amplitude of N2, P2, and N3 had not improved obviously compared with the first day after the surgery (Figure 2D3). The fundus blood vessels were normal and the retina was flat, without bulging after 6 months (Figure 2).

## Treatment and prognosis

### Surgical method

**Figure 3:**
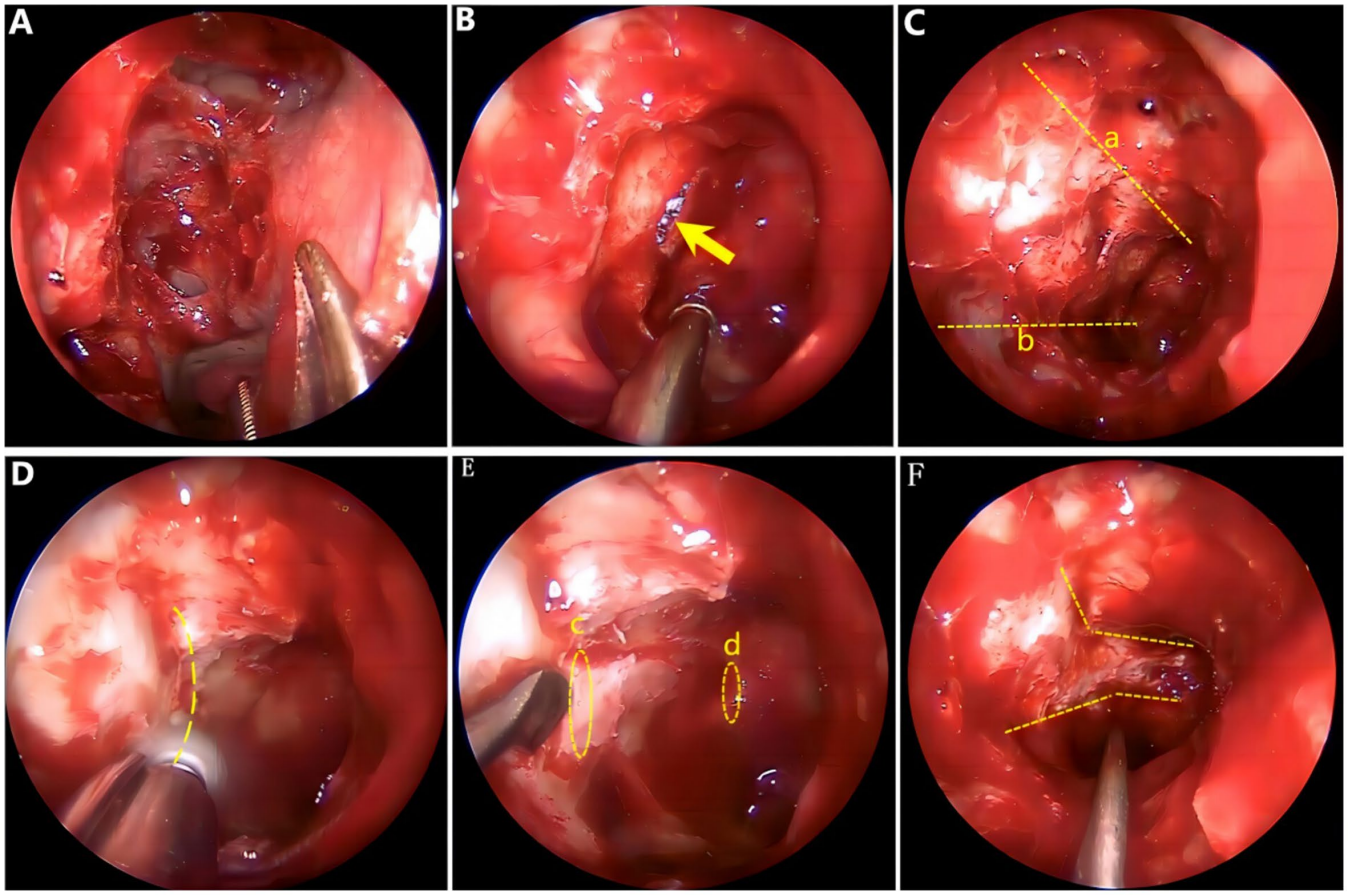
Surgical image. **(A)** Open ethmoid sinus and sphenoid sinus. **(B)** Fracture line of the optic nerve bony canal. **(C)** Orbital cranial line (a) and orbital floor line (b). **(D)** Thinning the bone of the optic nerve tube at 180°. **(E)** Starting point for bone removal: orbital ostia (c), end point for bone removal: cranial ostial (d). **(F)** Optic nerve tube decompressed adequately.

General anesthesia and supine position were used. (a) The deviated nasal septum that impeded the surgical field was corrected initially. (b) High-definition nasal endoscopy was used to open the maxillary sinus, ethmoid sinus, and sphenoid sinus according to the Messerklinger procedure. (c) The optic tube carina (part of the optic nerve) was confirmed to be free from the sphenoid sinus lumen, and the internal carotid artery carina was exposed, revealing the medial and inferior wall of the orbit and optic nerve tube. (d) Multiple fractures of the neural tube wall and multiple bone defects in the lower orbital wall were observed. (e) Part of the medial orbital wall and the inner as well as lower and upper inner walls of the optic nerve tube were thinned carefully, and a thin bone piece was peeled off carefully with a microstripper. (f) The full length of the optic nerve tube was opened and decompressed by approximately 180°. A gelatin sponge soaked with dexamethasone was covered over the optic nerve and an inflated sponge tamponade was used to compress the middle nasal passage to stop bleeding. Intraoperative endoscopic images are shown in Figure 3. After surgery, symptomatic comprehensive treatment, such as antibiotics, glucocorticoids, neurotrophic and vasodilators that can penetrate the blood and brain barrier, was administered. A total of nine patients experienced an improvement in their visual power, whereas four patients failed to show any improvement, resulting in a postoperative effective rate of 69.2%. The time from trauma to surgery ranged from 0.7 to 30 days (average time 5.7 ± 8.43 days). A total of 10 patients were operated on within 7 days, seven of whom showed improved visual power, resulting in an effective rate of 70%. Another three patients were operated on 7 days later, two of whom showed improved visual power, resulting in an effective rate of 66.7%. Only six patients had no light preoperatively, three of whom had improved visual power after surgery, resulting in an effective rate of 50.0%. A total of seven patients had residual light sensation preoperatively, six of whom had improved visual power after surgery, resulting in an effective rate of 85.7%. The VA of three cases was >0.02, and the VA improved in two cases after surgery (Table 2).

**Table 2:** Clinical features for operative efficiency.

Clinical features	Cases (*n*) (Operative efficiency (%))
Total cases	13 (69.2)
Gender	
Male	12 (58.3)
Female	1 (100)
Operation time	
≤ 7 days	10 (70)
>7 days	3 (66.7)
Initial vision	
No light	6 (50.0)
Residual light	7 (85.7)

## Discussion

TON is a rare but serious complication of closed head injury (Dhaliwal et al., 2016). The risk group is mainly young men aged 30 years range (Lee et al., 2010). Road traffic accidents (especially collisions with motorcycles, electric bicycles, and rickshaws), falls from heights, and other blunt trauma are the main causes of TON (Pirouzmand, 2012).

TON includes both primary and secondary injuries (Huempfner-Hierl et al., 2015). Primary injury is caused by transient external force resulting in optic nerve rupture, optic nerve contusion, or optic nerve sheath hemorrhage, leading to immediate vision loss. Secondary injury after external force can cause optic nerve edema, local vascular compression, vascular occlusion, and other circulatory disorders, which can lead to avascular necrosis of the optic nerve, resulting in delayed vision loss, which usually occurs within hours or days after injury. The optic nerve is divided into intrabulbar, intraorbital, intracanal, and intracranial segments, of which the main rhinological segments related to rhinology are the intraorbital and intracanal segments. Approximately 90% of traumatic optic neuropathy cases occur in the intracanal segment (Steinsapir and Goldberg, 2011). Orbital thin-slice CT scan can determine the location of optic nerve tube injury effectively. A quick eye examination after an injury can assess the patient’s vision. Initial fundoscopy can also help rule out pre-existing optic neuropathy and retinopathy, and might identify patients with papillary swelling and adjacent retinal hemorrhage. Visual Evoked Potential (VEP) is an objective indicator of optic nerve function and can be used as a predictor of long-term prognosis, and its reduced amplitude and increased latency are associated closely with visual impairment (Tabatabaei et al., 2011).

In recent years, there has still been no unified standard treatment strategy for clinicians to consider for TON. Surgical intervention or glucocorticoid pulse therapy is an effective treatment option in patients with TON (Simsek et al., 2006), although the exact efficacy of optic nerve decompression remains controversial. There are some opinions suggesting that optic nerve decompression improves prognosis, emphasizing the importance of surgery (He et al., 2016). The operation improves vision by opening the full length of the optic nerve bone canal, resecting 1/2 of the circumference of the tube wall, and cutting the optic nerve sheath and the total tendon ring at the front end longitudinally (Sofferman, 1981). The operation improves vision by opening the full length of the optic nerve bone canal, resecting 1/2 of the circumference of the tube wall, and cutting the optic nerve sheath and the total tendon ring at the front end longitudinally (Dhaliwal et al., 2016). By contrast, some studies suggest that optic nerve decompression should not be recommended for patients owing to the fact that surgical decompression of the optic nerve does not result in good clinical benefits, and that surgery itself is a traumatic procedure, coupled with the risks associated with surgery (Sosin et al., 2016). Furthermore, the effect of optic nerve decompression surgery is affected largely by relevant prognostic factors, such as the patient’s consciousness after injury and appropriate operation time (Ma et al., 2018).

In this study, 13 patients with TON were assessed, all of whom underwent transnasal endoscopic optic nerve decompression, and the visual improvement of patients before and after surgery was analyzed comparatively; 69.2% of patients had an improvement in VA postoperatively. This suggests that optic nerve decompression has a significant effect on the treatment of TON. To investigate the influence of related prognostic factors, our study divided patients into two groups based on the time of onset to surgery, with one group being within 7 days and the other being >7 days, and analyzed the effective rate of postoperative VA improvement between the two groups. The results showed that the effective rate of visual improvement in patients within 7 days was better than the patients who took more than 7 days to receive treatment. This indicates that the timing of TON surgery is related closely to the pathophysiology of optic nerve injury. Shortening the timing of surgery as much as possible is beneficial in interrupting the progression of pathophysiological processes and minimizing the harm caused by secondary injury to the optic nerve. Although surgery has been reported to improve impaired vision after months of visual impairment (Thakar et al., 2003), most of the center’s findings suggest that early surgical intervention is still performed within 7 or even 3 days of visual impairment (Chen et al., 2018).

Studies have demonstrated that the rate of visual improvement after surgery in patients with TON who have residual vision is significantly higher than that of those with non-photosensitive TON (Ma et al., 2018). This study further revealed that the rate of postoperative visual improvement in TON patients without LP before surgery was significantly lower than that in patients with residual vision. This might be attributed to the fact that patients with totally blind TON suffer irreversible optic neuropathological changes and have fewer retinal ganglion cells (RGCs) than those with residual vision. Nevertheless, preoperative VA and timing are sometimes not isolated and correlated, thus the effects of both should be taken into account when undertaking surgery in patients with TON.

The impact of optic nerve fractures on the final visual outcome of patients with TON remains a matter of debate. Studies have demonstrated that the presence of optic nerve fractures is an independent predictor of poor visual prognosis (Yu et al., 2018). Nevertheless, some reports suggest that the presence or absence of optic nerve fractures does not influence the treatment outcome of patients with TON significantly (Gupta et al., 2007). The use of glucocorticoids in the treatment of TON has also been a subject of debate. There is no uniform standard for the treatment of TON, and its application is based on the treatment of acute spinal cord injury (Bracken et al., 1997). The purpose of glucocorticoid therapy for TON is to reduce optic nerve microcirculation spasm, anti-inflammatory effects, reduce swelling, scavenge free radicals, and protect the optic nerve by antioxidants. Entezari et al. (2007) found no significant difference between the glucocorticoid-based and non-glucocorticoid groups, whereas Kircher et al. (2008) have suggested that glucocorticoids can promote the repair of damaged optic nerves. Taking into account its anti-inflammatory, swelling and other effects, as well as the serious consequences of TON, the leading clinical recommendation is to apply glucocorticoids as soon as possible after the onset, while avoiding adverse reactions, such as gastrointestinal ulcers, osteoporosis, and elevated blood glucose. All patients in this study were treated with glucocorticoids after surgery.

## Conclusion

In conclusion, this study found that transnasal endoscopic optic nerve decompression is a safe and effective treatment for TON. Preoperative residual vision and surgery within 7 days are essential for successful outcomes, but should not be strictly adhered to, and the earlier surgery is performed, the better the prognosis, depending on the pathophysiology of the optic nerve injury. Additionally, owing to the involvement of multiple disciplines, such as ophthalmology, otolaryngology, and neurosurgery, there is a disparity in the understanding of the diagnosis and treatment of the disease. Therefore, interdisciplinary collaboration is necessary to develop the most suitable treatment plan for the patient.

## Data availability statement

The original contributions presented in the study are included in the article/supplementary material, further inquiries can be directed to the corresponding authors.

## Ethics statement

Written informed consent was obtained from the individual(s), and minor(s)’ legal guardian/next of kin, for the publication of any potentially identifiable images or data included in this article.

## Author contributions

XT and CX designed and conducted the study. XT, CX, HQ, YO, and JR collected, analyzed, managed, and interpreted the data. XT and HQ wrote the manuscript and conducted the statistical analysis. YS, YF, and GL made key revisions to the manuscript. All authors contributed to the article and approved the submitted version.

## Funding

This study was supported by Science, Technology and Innovation Commission of Shenzhen Municipality under Grant (number GJHZ20190821113605296).

## Conflict of interest

The authors declare that the research was conducted in the absence of any commercial or financial relationships that could be construed as a potential conflict of interest.

## Publisher’s note

All claims expressed in this article are solely those of the authors and do not necessarily represent those of their affiliated organizations, or those of the publisher, the editors and the reviewers. Any product that may be evaluated in this article, or claim that may be made by its manufacturer, is not guaranteed or endorsed by the publisher.
